# Overexpression of AKR1B10 Predicts Poor Prognosis in Gastric Cancer Patients Undergoing Surgical Resection

**DOI:** 10.3390/curroncol30010007

**Published:** 2022-12-21

**Authors:** Yu-Yin Liu, Yueh-Wei Liu, Gong-Kai Huang, Kuo-Chen Hung, Yu-Hung Lin, Cheng-Hsi Yeh, Shih-Min Yin, Ching-Hua Tsai, Yen-Hao Chen

**Affiliations:** 1 Division of General Surgery, Department of Surgery, Kaohsiung Chang Gung Memorial Hospital and Chang Gung University College of Medicine, Kaohsiung 833, Taiwan; 2 Department of Anatomic Pathology, Kaohsiung Chang Gung Memorial Hospital and Chang Gung University College of Medicine, Kaohsiung 833, Taiwan; 3 Division of Trauma Surgery, Department of Surgery, Kaohsiung Chang Gung Memorial Hospital and Chang Gung University College of Medicine, Kaohsiung 833, Taiwan; 4 Division of Hematology-Oncology, Department of Internal Medicine, Kaohsiung Chang Gung Memorial Hospital and Chang Gung University College of Medicine, Kaohsiung 833, Taiwan; 5 School of Medicine, College of Medicine, Chang Gung University, Taoyuan 333, Taiwan; 6 School of Medicine, Chung Shan Medical University, Taichung 402, Taiwan; 7 Department of Nursing, School of Nursing, Fooyin University, Kaohsiung 831, Taiwan

**Keywords:** AKR1B10, gastric cancer, survival, adjuvant, signet ring cell

## Abstract

Aldo–keto reductase family 1 member B10 (AKR1B10) is associated with several cancers, but the prognostic role in gastric cancer (GC) remains unclear. We enrolled 359 GC patients who underwent a gastrectomy with D2 lymph node dissection. AKR1B10 expression was scored using an immunoreactive scoring system based on immunohistochemistry. Adjuvant chemotherapy with S-1 or oxaliplatin plus capecitabine was administered to pathological stage II or III disease patients. There were 117 (32.6%) and 242 (67.4%) patients with AKR1B10 overexpression and low expression, respectively. Patients overexpressing AKR1B10 had worse 5-year disease-free survival (DFS) and overall survival (OS) rates than those with low expression of AKR1B10. Pathological T3–T4 stage, pathological stage III, lymph node ratio ≥25%, and AKR1B10 overexpression were independent prognostic factors for worse DFS and OS in univariate and multivariate analyses. For 162 stage II or III patients who received adjuvant chemotherapy after surgical resection and 59 patients with signet ring cell carcinoma histology, AKR1B10 overexpression was also associated with inferior DFS and OS. AKR1B10 was not associated with clinical survival in stage I GC patients. In conclusion, AKR1B10 overexpression may be an independent prognostic factor for worse survival in GC patients who underwent gastrectomy with D2 lymph node dissection.

## 1. Introduction

Gastric cancer (GC) is one of the most aggressive malignancies worldwide and is the 8th leading cause of cancer-associated mortality in Taiwan [1]. For operable diseases, radical gastrectomy using extended regional lymph node dissection is the gold standard treatment. Recently, adjuvant chemotherapy has been approved to improve disease progression and decrease tumor recurrence [2,3]. Despite the development of surgical techniques, chemotherapy, radiotherapy, and immunotherapy, the long-term survival rate still remains poor. This indicates that identifying novel mechanisms to improve clinical outcomes is crucial.

The aldo–keto reductase protein superfamily consists of over 190 members and is a family of enzymes that includes many related monomeric NADPH-dependent oxidoreductases [4]. These enzymes reduce carbonyl substrates such as sugar aldehydes, keto-steroids, keto-prostaglandins, retinals, quinones, and lipid peroxidation byproducts. To date, 15 human aldo–keto reductases (AKRs) belonging to three different AKR families are known: 10 in the AKR1, 3 in AKR6, and 2 in AKR7. Increasing evidence suggests that they are involved in cancer development, progression, and treatment [4].

Aldo–keto reductase family 1 member B10 (AKR1B10) was initially identified in liver cancer [5]. It is highly expressed in the small intestine, colon, and adrenal gland [6,7]. Of note, its expression has also been found in cancers of the liver, breast, endometrium, cervix, and lung [5,8,9,10,11,12,13,14]. Previous studies have shown that AKR1B10 may contribute to tumor progression through several mechanisms. First, AKR1B10 can effectively detoxify reactive carbonyl species (RCS) [15]. These are highly electrophilic and cytotoxic; thus, their removal by AKR1B10 would facilitate tumor cell growth. Second, AKR1B10 efficiently converts retinals such as all-*trans*-retinal, 9-*cis*-retinal, and 13-*cis*-retinal to their corresponding retinols. The retina is a precursor of retinoic acid. The conversion of retinal to retinol results in the suppression of this conversion of retinal to retinoic acid, which is a major active anti-neoplastic metabolite. Retinoic acid binds to retinoid X nuclear receptors and heterodimerizes with peroxisome proliferator-activated receptor (γ). This step regulates target gene transcription through the peroxisome proliferator response element, leading to an antiproliferative response and cell differentiation. The reductive activity of retinals to retinols by AKR1B10 deprives retinoic acid receptors access to their ligands. This results in cell growth and blockage of differentiation, and apoptosis; these events aid in the multi-step carcinogenic process [6,16]. Third, recent studies have shown that AKR1B10 is implicated in the carcinogenic activation of polycyclic aromatic hydrocarbons induced by tobacco smoke and thus, might be involved in tobacco-related carcinogenesis [8,17]. Fourth, AKR1B10 is thought to contribute to chemoresistance in various cancers [18,19,20]. One of the possible mechanisms is that many chemotherapeutic agents contain active carbonyl groups, such as doxorubicin or daunorubicin. AKR1B10 could reduce the carbonyl group to less toxic alcoholic forms, thus inducing drug resistance to chemotherapy [18]. Fifth, AKR1B10 also functions as an important regulator of de novo fatty acid/lipid synthesis, which is essential for cancer cell growth and division. AKR1B10 prevents ubiquitination and proteasomal degradation of acetyl-CoA carboxylase-α (ACACA), a rate-limiting enzyme in de novo fatty acid synthesis, thus promoting fatty acid synthesis [21,22]. Therefore, AKR1B10 could play an important role in various aspects of tumor progression and may be a potential therapeutic target for cancer treatment.

However, the role of AKR1B10 in GC remains unclear. AKR1B10 is regarded as a tumor suppressor. Its knockdown could inhibit tumor proliferation and migration by enhancing epithelial–mesenchymal transition (EMT) in vivo. Furthermore, a high expression of AKR1B10 was significantly associated with a better 5-year survival rate [23,24]. In contrast, GC patients with overexpression of AKR1B10 were found to have advanced lymph node metastasis, fewer tumor regression, and worse overall survival (OS) than those without AKR1B10 expression [25]. As stated above, a meta-analysis reported by Liu et al. demonstrated that high expression of AKR1B10 was not related to disease-free survival (DFS) and OS [26]. Therefore, the current study includes the largest sample size to date. It aimed to elucidate the clinical impact of AKR1B10 in GC patients undergoing surgical resection.

## 2. Materials and Methods

### 2.1. Patients

We retrospectively reviewed patients with GC who underwent surgical resection at the Kaohsiung Chang Gung Memorial Hospital, between January 2011 and December 2016. The following enrollment criteria were considered: (i) presence of adenocarcinoma; other histological types, such as a neuroendocrine tumor, gastrointestinal stromal tumor, or squamous cell carcinoma were excluded; (ii) GC patients who underwent radical total or subtotal gastrectomy with D2 lymph node (LN) dissection, palliative gastrectomy, D1 LN dissection, or no LN dissection; (iii) pathological stage I–III, based on the 8th edition of the American Joint Committee on Cancer (AJCC) staging system [27]; (iv) Eastern Cooperative Oncology Group (ECOG) 0–1; (v) adjuvant chemotherapy with S-1 or oxaliplatin/capecitabine was prescribed if indicated [2,3]; and (vi) distant metastasis or history of second primary malignancy. In total, 359 GC patients who met these inclusion criteria were included in our study.

Post-operative patients were followed up at the outpatient clinic every 2–4 weeks during the first year. Each patient underwent computed tomography (CT) of the abdomen every 3–6 months; a panendoscopy was performed every 6 months for at least one year. Adjuvant chemotherapy with S-1 or oxaliplatin/capecitabine was administered to patients with pathological stage II or III. The duration and dose of chemotherapy were based on previous clinical trials [2,3]. The definition of LN ratio is the total number of involved LNs divided by the dissected LNs [28].

### 2.2. Immunohistochemistry

The formalin-fixed paraffin-embedded surgical specimen of each patient was sectioned to be 4 μm thick. Next, the deparaffinization step was performed in a dry oven at 60 °C for one hour. Antigen retrieval with 0.01 mol/L citrate buffer (pH 6.0) in a hot water bath (95 °C) for 20 min, and peroxidase blocking using 0.3% hydrogen peroxide for five minutes was done. The specimen was then incubated with the AKR1B10 antibody (Abnova, H00057016-M01, 1:80, Walnut, CA, USA) for 30 min at room temperature. A healthy human colon was used as a positive control, and the negative control was performed by replacing the primary antibody with an isotype-matched irrelevant antibody. These slides were independently investigated by two pathologists (S.L. Wang and W.T. Huang) in a blinded manner. The immunohistochemical staining was performed according to a previously published method [29].

**Figure 1:**
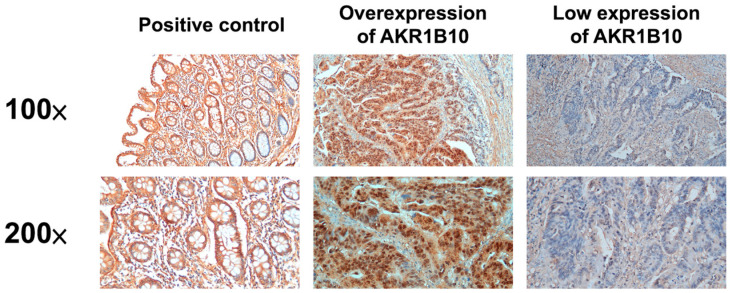
The immunohistochemical analysis of AKR1B10 in gastric cancer patients who received gastrectomy with D2 lymph node dissection.

The expression of AKR1B10 was scored based on the immunoreactive score (IRS) system. It provides a range of 0–12 as a product of multiplication between the staining intensity score (0: no staining, 1: weak, 2: moderate, and 3: strong) and positive cell proportion score (0:0%, 1:1–10%, 2:11–50%, 3:51–80%, and 4:81–100%) [30]. Any specimen with a sum score >6 was defined as overexpression of AKR1B10. The expression levels of AKR1B10 are shown in Figure 1.

### 2.3. Statistical Analysis

Statistical analyses were conducted using SPSS software (version 26.0; International Business Machines Corp., New York, NY, USA) and R software version 3.3.0 (R foundation for Statistical Computing, Vienna, Austria). The differences in categorical variables between groups were examined using the chi-square test. DFS was defined as the duration from the date of GC diagnosis to death or last living contact. OS was calculated from surgery to tumor recurrence or death from any cause. Univariate analysis was performed using the Kaplan–Meier method, and the log-rank test was used to examine potential differences. All categorical variables (*p*-values < 0.1) in the univariate analyses were further entered into a multivariate Cox proportional hazards model to identify significant factors using forward stepwise selection independently. Statistical significance was defined as a two-tailed *p*-value < 0.05.

### 2.4. Construction and Validation of Nomogram

A nomogram to predict rate of recurrence or death was constructed based on the significant factors of the multivariate analyses. An external independent validation cohort was used to validate the nomogram, and the predictive performance was evaluated by the concordance index (C-index) and calibration plot. The measurement of nomogram between performance and predicted outcomes was determined by C-index. The comparison between actual and nomogram-predicted outcome was performed by calibration plots using a 45-degree line as an optimal model.

### 2.5. Ethical Statement

The study was conducted in accordance with the Declaration of Helsinki and was approved by the Institutional Review Board of Chang Gung Medical Foundation (201900004B0). Written informed consent was not required because of the retrospective design of this study.

## 3. Results

### 3.1. Patient Characteristics

A total of 359 GC patients, who underwent a gastrectomy with D2 lymph node dissection, were identified upon a retrospective review of our database. In total, 238 male and 121 female patients with a median age of 66 years (range: 30–89 years) were selected. The location of gastric tumors showed cardia/fundus in 34 (9.5%), body in 134 (37.3%), antrum in 178 (49.6%), and pylorus in 13 (3.6%) patients. With respect to the surgical procedures, 44 (12.3%) underwent total gastrectomy, 292 (81.3%) underwent subtotal gastrectomy, and 23 (6.4%) patients underwent proximal gastrectomy. Regarding pathological T status, 109 (30.4%) were T1, 49 (13.6%) were T2, 87 (24.2%) were T3, and 114 (31.8%) were T4. Furthermore, the pathological N status was N0 in 107 (47.4%), N1 in 42 (11.7%), N2 in 56 (15.6%), and N3 in 91 (25.3%) patients. Moreover, 127 (35.4%), 82 (22.8%), and 150 (41.8%) patients were diagnosed with pathologic stages I, II, and III, respectively. Analysis of tumor grade included grade 1 in 28 (7.8%), grade 2 in 111 (30.9%), and grade 3 in 220 (61.3%) patients. In addition, there were 175 (48.7%) patients with perineural invasion (PNI) and 184 (51.3%) without PNI; 199 (55.4%) patients had lymphovascular invasion (LVI), and the remaining 160 (44.6%) had no LVI. The median follow-up period was 46.9 months (range: 1.3–109.5 months). The clinicopathological characteristics of the 359 GC patients are shown in Table 1.

### 3.2. AKR1B10 Expression and Clinical Outcome

There were 117 (32.6%) and 242 (67.4%) patients who showed overexpression and low AKR1B10 expression, respectively. The baseline characteristics between these two groups did not differ significantly; these included age, sex, pathological T status, pathological N status, pathological stage, tumor grade, PNI status, LVI status, and LN ratio, except for the surgical procedure. However, there was a higher percentage of total gastrectomy in the AKR1B10 overexpression group than in the low AKR1B10 overexpression group (20.5% vs. 8.3%, *p* = 0.001). The clinicopathological characteristics of both groups are shown in Table 2.

**Figure 2:**
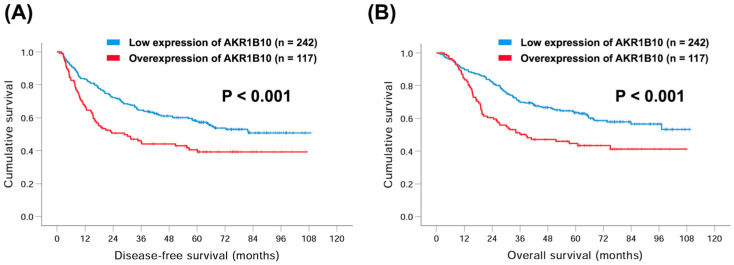
Kaplan–Meier curves comparing 5-year disease-free survival (DFS) and overall survival (OS) rates in GC patients with overexpression or low expression of AKR1B10. (**A**) DFS; (**B**) OS.

Regarding DFS, no significant differences in age or sex were noted in both univariate and multivariate analyses. However, the univariate analysis demonstrated that a worse 5-year DFS rate was found in patients with pathological T3–T4 (compared to pathological T1–T2, 34.5% versus 74.7%, *p* < 0.001), pathological N1–N3 (compared to pathological N0, 35.3% versus 71.0%, *p* < 0.001), pathological stage III (compared to pathological stage I–II, 25.9% versus 70.9%, *p* < 0.001), tumor grade 3 (compared to tumor grade 1–2, 48.2% versus 58.5%, *p* = 0.049), total gastrectomy (compared to no total gastrectomy, 37.6% versus 54.3%, *p* = 0.014), PNI (compared to no PNI, 32.0% versus 71.1%, *p* < 0.001), LVI (compared to no LVI, 39.4% versus 68.3%, *p* < 0.001), LN ratio ≥ 25% (compared to LN ratio < 25%, 15.6% versus 64.1%, *p* < 0.001), and overexpression of AKR1B10 (compared to low expression of AKR1B10, 40.5% versus 57.8%, *p* = 0.001, Figure 2A). Multivariate analysis further revealed that pathological T3–T4 (hazard ratio (HR): 2.08, 95% confidence interval (CI): 1.36–3.17, *p* = 0.001), pathological stage III (HR: 1.62, 95% CI: 1.02–2.59, *p* = 0.043), LN ratio ≥ 25% (HR: 2.22, 95% CI: 1.49–3.32, *p* < 0.001), and overexpression of AKR1B10 (HR: 1.81, 95% CI: 1.32–2.47, *p* < 0.001) were independent prognostic factors of worse DFS.

For OS, the differences in age, sex, and tumor grade between the two groups were not significant. Based on the univariate analysis, patients with pathological T3–T4 (compared to pathological T1–T2, 40.2% versus 79.4%, *p* < 0.001), pathological N1–N3 (compared to pathological N0, 40.3% versus 76.5%, *p* < 0.001), pathological stage III (compared to pathological stage I–II, 28.2% versus 77.9%, *p* < 0.001), total gastrectomy (compared to no total gastrectomy, 41.9% versus 59.7%, *p* = 0.006), PNI (compared to no PNI, 35.5% versus 77.5%, *p* < 0.001), LVI (compared to no LVI, 43.5% versus 74.9%, *p* < 0.001), LN ratio ≥ 25% (compared to LN ratio < 25%, 18.2% versus 70.0%, *p* < 0.001), and overexpression of AKR1B10 (compared to low expression of AKR1B10, 44.7% versus 63.5%, *p* < 0.001, Figure 2B) were found to have worse 5-year OS rate. Moreover, pathological T3–T4 (HR: 1.97, 95% CI: 1.24–3.12, *p* = 0.004), pathological stage III (HR: 1.83, 95% CI: 1.12–3.00, *p* = 0.017), LN ratio ≥ 25% (HR: 2.23, 95% CI: 1.48–3.35, *p* < 0.001), and overexpression of AKR1B10 (HR: 1.93, 95% CI: 1.39–2.67, *p* < 0.001) were independent prognostic factors of worse OS in the multivariate analysis. The detailed presentation of univariate and multivariate analyses was shown in Table 3 and Table 4. 

### 3.3. Subgroup Analyses of AKR1B10 and Survival

**Figure 3:**
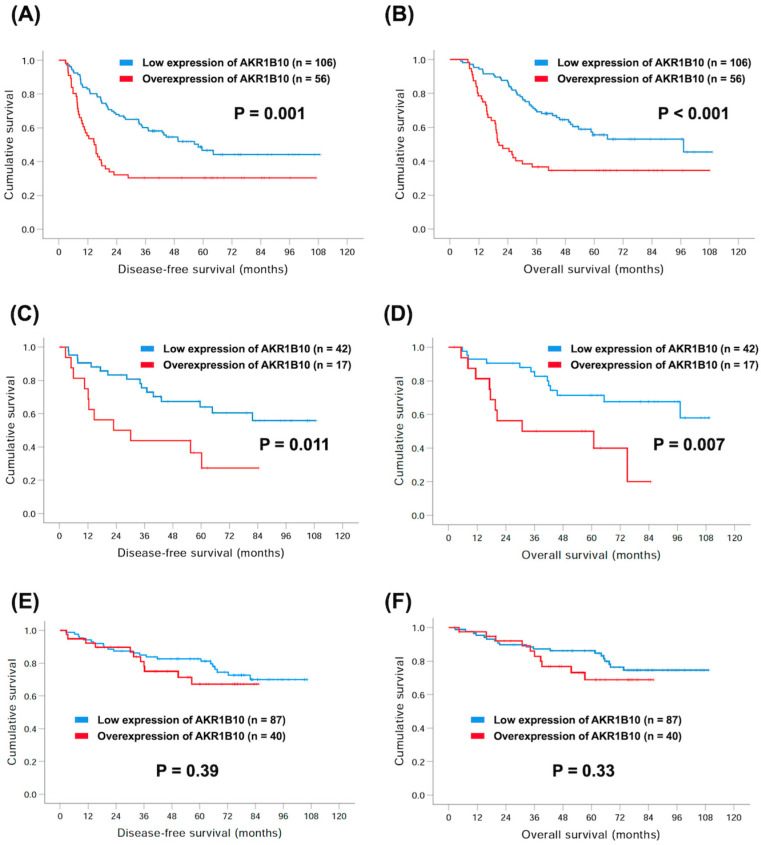
Comparison of Kaplan–Meier curves of 5-year DFS and OS rates between GC patients with overexpression and low expression of AKR1B10. Adjuvant chemotherapy subgroup: (**A**) DFS and (**B**) OS; signet ring cell carcinoma subgroup: (**C**) DFS and (**D**) OS; pathological stage I subgroup: (**E**) DFS and (**F**) OS.

Among these 359 GC patients, 162 with stage II or stage III GC received adjuvant chemotherapy after surgical resection, including S-1 or oxaliplatin plus capecitabine. There were 56 (34.6%) and 106 (65.4%) patients with overexpression and low expression of AKR1B10, respectively. The worse 5-year DFS (30.4% versus 46.6%, *p* = 0.001, Figure 3A) and 5-year OS rates (34.6% versus 63.5%, *p* < 0.001, Figure 3B) were observed in patients with overexpression of AKR1B10 compared to those with low expression of AKR1B10.

On the other hand, there were a total of 59 GC patients with signet ring cell (SRC) carcinoma in our cohort study, including 17 (28.8%) and 42 (71.2%) patients with overexpression and low expression of AKR1B10, respectively. Patients overexpressing AKR1B10 had lower 5-year DFS (36.5% vs. 64.0%, *p* = 0.011, Figure 3C) and 5-year OS rates (50.0% vs. 71.4%, *p* = 0.007, Figure 3D) than those with low expression of AKR1B10.

However, for 127 patients with stage I disease, including 40 (31.5%) patients with overexpression of AKR1B10 and 87 (68.5%) with low expression of AKR1B10, the difference between the groups did not reach significance. This was despite inferior 5-year DFS (67.2% versus 81.2%, *p* = 0.39, Figure 3E) and 5-year OS rates (68.8% versus 86.1%, *p* = 0.33, Figure 3F), in addition to overexpression of AKR1B10 in comparison with those with low expression, in these patients.

### 3.4. Nomogram and Validation

**Figure 4:**
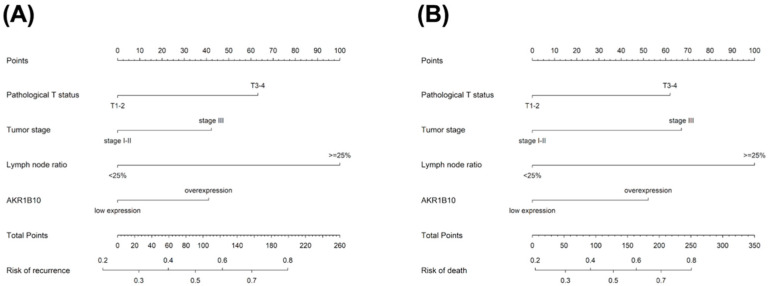
Nomogram predicting the risk of recurrence (**A**) and death (**B**). For each patient, corresponding clinicopathological feature points were calculated and summed up to obtain total points. Predicted recurrence and death could be estimated based on total points for each patient.

**Figure 5:**
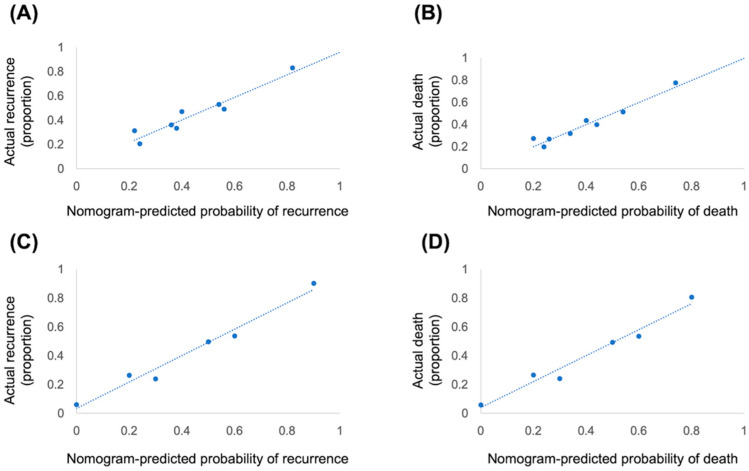
Calibration plots show the relationship between the predicted probabilities based on the nomogram and actual values of the training cohort (**A**,**B**) and validation cohort (**C**,**D**).

The nomogram that integrated four independent risk factors to predict recurrence and death in the training cohort is shown in Figure 4. The C-index for the model prediction was 0.75 (95% CI: 0.70–0.80) for recurrence and 0.76 (95% CI: 0.71–0.81) for death in the training model. The calibration plot for the recurrence and death revealed an optimal agreement between the prediction by the nomogram and actual observation (Figure 5A,B). An additional 47 patients with gastric cancer who underwent radical gastrectomy from January 2017 to December 2018 were independently identified to validate this nomogram. The validation cohort demonstrated an ideal correlation with the actual recurrence (C-index 0.86; 95% CI: 0.78–0.97) and actual death (C-index 0.84; 95% CI: 0.73–0.95). Moreover, the external calibration plot for the recurrence and death also revealed good agreement (Figure 5C,D).

## 4. Discussion

Gastric cancer is an aggressive malignancy; radical gastrectomy with extended regional lymph node dissection is the gold standard treatment for operable diseases. In addition to the well-known risk factors, such as advanced tumor stage or higher LN ratio, several predictive biomarkers have been identified in previous studies [25,31]. AKR1B10 has been reported to be associated with cancer development, progression, and treatment through several mechanisms, such as detoxification of RCS or regulation of lipid synthesis [4]. AKR1B10 is expressed in many organs and it has also been studied in a variety of cancers, such as in liver, lung, gynecological cancer, and breast [5,8,9,10,11,12,13,14]. Moreover, AKR1B10 positively correlated with tumor size, lymph node metastasis, and clinical outcome in breast cancer. Silencing of AKR1B10 also inhibited tumor proliferation in cell lines and animal studies. In addition, the serum AKR1B10 was higher in patients with breast cancer than in the healthy cohort. In our study, poor clinical outcomes were noted in patients with advanced pathological T or N status, tumor stage, total gastrectomy, PNI, LVI, and a higher LN ratio. In addition, 117 (32.6%) patients were found to overexpress AKR1B10; univariate and multivariate analyses showed that this overexpression was an independent prognostic factor for worse DFS and OS in GC patients.

However, the role of AKR1B10 remains unclear. Growing evidence has shown that AKR1B10 is a tumor suppressor [23,24]. Shao et al. reported that AKR1B10 expression was significantly related to smaller tumor size, lower depth of invasion, negative lymph node metastasis, negative venous invasion, and advanced tumor stage [23]. In addition, AKR1B10 knockdown increased tumor cell proliferation, whereas its overexpression reduced tumor growth by enhancing EMT in an in vitro study. Depletion of AKR1B10 could facilitate tumor cell proliferation, resulting in tumor size progression and body weight gain in an in vivo xenograft model [23]. Another GC study demonstrated that the expression of AKR1B10 mRNA was higher in normal tissues than in GC tissues [24]. The OS was significantly higher in patients with positive AKR1B10 expression than in those with negative AKR1B10 expression. Its expression was an independent prognostic factor of better survival in Cox regression analysis [24]. In contrast, GC patients with AKR1B10 overexpression had advanced lymph node metastasis, fewer tumor regression, and worse overall survival (OS) compared to those with negative expression of AKR1B10 [25]. Its expression was also correlated with better tumor regression for patients receiving neoadjuvant chemotherapy. A meta-analysis further revealed that high expression of AKR1B10 was not found related to DFS and OS [26]. In our study, we enrolled a large sample size and tried to elucidate the clinical impact of AKR1B10 in GC patients receiving surgical resection. We found that overexpression of AKR1B10 was an independent prognostic factor of worse DFS and OS in the univariate and multivariate analyses. This indicated that AKR1B10 might be regarded to possess an oncogenic role in GC patients. In addition, the prognostic role of AKR1B10 was also validated in TCGA database. In this gastric adenocarcinoma cohort from TCGA database, there were no significant differences of OS between overexpression and low expression of AKR1B10 in whole population; however, in the subgroup analysis, the survival outcome in difference races is a little different, better OS was mentioned in Asian patients with low expression of AKR1B10 compared to Asian patients with overexpression of AKR1B10. On the other hand, the expression of AKR1B10 increased with a trend from early stage to advanced stage, and a similar finding was also mentioned in the status of lymph node metastasis [32].

Adjuvant chemotherapy has been proven to be a standard treatment for stage II or stage III GC patients who underwent surgical resection [33,34]. A previous study showed that AKR1B10 expression could predict the response to neoadjuvant chemotherapy in GC patients (25). Better tumor regression was found to be higher in patients with negative AKR1B10 expression than in those with positive AKR1B10 expression (60% versus 40%, *p* = 0.033). However, the role of AKR1B10 in adjuvant therapies remains unclear. In our study, we investigated the role of AKR1B10 in GC patients who received adjuvant chemotherapy after surgery. In 162 patients who received S-1 or oxaliplatin plus capecitabine as adjuvant chemotherapy, overexpression of AKR1B10 was strongly associated with worse 5-year DFS (30.4% vs. 46.4%, *p* = 0.001) and OS (34.6% vs. 63.5%, *p* < 0.001) rates. This strongly suggests that AKR1B10 may be regarded as a poor prognostic factor in GC patients undergoing adjuvant chemotherapy.

SRC carcinoma is a rare subtype of gastric adenocarcinoma in which cells present a large vacuole in histology. It is different from conventional GC to some degree; for example, its higher prevalence in younger age, location in the upper third stomach, and presence of higher number of Borrmann type IV tumors. Growing evidence has demonstrated that SRC carcinoma is associated with poor survival outcome [35,36,37]. There were 59 (16.4%) patients with SRC carcinoma in our cohort, and AKR1B10 expression was still related to clinical outcomes. Patients overexpressing AKR1B10 were found to have worse 5-year DFS (36.5% vs. 64.0%, *p* = 0.011) and OS (50.0% vs. 71.4%, *p* = 0.007) rates than those with low AKR1B10 expression. However, the prognostic value of AKR1B10 in early-stage GC remains unclear. We enrolled 127 stage I GC patients, including 40 with overexpression of AKR1B10 and 87 with low AKR1B10 expression. Patients overexpressing AKR1B10 had relatively inferior 5-year DFS (67.2% versus 81.2%) and OS (68.8% versus 86.1%) rates than those with low expression of AKR1B10. However, the difference between the two groups did not differ significantly, indicating that the role of AKR1B10 may not be significant in stage I GC.

Our study had some limitations. First, because of the retrospective design and the fact that all patients were treated at a single institution which may have led to a selection bias. For example, GC patients without surgical specimens for analysis were not enrolled in our study. Second, there was a higher percentage of total gastrectomy in patients overexpressing the AKR1B10 group compared to those with a lower expression (20.5% versus 8.3%). Although total gastrectomy was a prognostic factor for worse DFS and OS in univariate analysis, it was not significant in the multivariate analysis. This indicates that AKR1B10-related poor prognosis may not be completely associated with this reason but owing to its oncogenic mechanisms. However, to the best of our knowledge, the current study constitutes the largest analysis that explores the prognostic value of AKR1B10 in GC patients who underwent gastrectomy with D2 lymph node dissection. It may provide more evidence for further basic research.

## 5. Conclusions

The results of our study confirm that overexpression of AKR1B10 may be regarded as an independent prognostic factor for GC patients with worse survival and who underwent gastrectomy with D2 lymph node dissection.

## Figures and Tables

**Table 1:** Characteristics of 359 patients with gastric adenocarcinoma receiving surgical resection.

Characteristics	Patient number (%)
Age (years)	66 years old (30–89)
Sex	
Male	238 (66.3%)
Female	121 (33.7%)
Location	
Cardia/Fundus	34 (9.5%)
Body	134 (37.3%)
Antrum	178 (49.6%)
Pylorus	13 (3.6%)
Pathological T status	
1	109 (30.4%)
2	49 (13.6%)
3	87 (24.2%)
4	114 (31.8%)
Pathological N status	
0	170 (47.4%)
1	42 (11.7%)
2	56 (15.6%)
3	91 (25.3%)
Pathological tumor stage	
I	127 (35.4%)
II	82 (22.8%)
III	150 (41.8%)
Grade	
1	28 (7.8%)
2	111 (30.9%)
3	220 (61.3%)
Perineural invasion	
Yes	175 (48.7%)
No	184 (51.3%)
Lymphovascular invasion	
Yes	199 (55.4%)
No	160 (44.6%)
Gastrectomy	
Total	44 (12.3%)
Subtotal	292 (81.3%)
Proximal	23 (6.4%)

**Table 2:** Comparison of clinicopathological parameters in 359 gastric adenocarcinoma patients receiving gastrectomy.

Characteristics	Overexpression of AKR1B10 (*n* = 117)	Low Expression of AKR1B10 (*n* = 242)	*p* Value
Age			0.84
<60 years	37 (31.6%)	74 (30.6%)	
≥60 years	80 (68.4%)	168 (69.4%)	
Sex			0.71
Male	76 (65.0%)	162 (66.9%)	
Female	41 (35.0%)	80 (33.1%)	
Pathological T status			0.91
1 + 2	52 (44.4%)	106 (43.8%)	
3 + 4	65 (55.6%)	136 (56.2%)	
Pathological N status			0.75
0	54 (46.2%)	116 (47.9%)	
1 + 2 + 3	63 (53.8%)	126 (52.1%)	
Pathological tumor stage			0.35
I + II	64 (54.7%)	145 (59.9%)	
III	53 (45.3%)	97 (40.1%)	
Grade			0.06
1 + 2	37 (31.6%)	102 (42.1%)	
3	80 (68.4%)	140 (57.9%)	
Total gastrectomy			0.001 *
Yes	24 (20.5%)	20 (8.3%)	
No	93 (79.5%)	222 (91.7%)	
Perineural invasion			0.07
Yes	65 (55.6%)	110 (45.5%)	
No	52 (44.4%)	132 (54.5%)	
Lymphovascular invasion			0.11
Yes	72 (61.5%)	127 (52.5%)	
No	45 (38.5%)	115 (47.5%)	
Lymph node ratio			0.06
<25%	81 (69.2%)	190 (78.5%)	
≥25%	36 (30.8%)	52 (21.5%)	

* Statistically significant.

**Table 3:** Univariate and multivariate analyses of disease-free survival (DFS) in 359 gastric adenocarcinoma patients receiving gastrectomy.

Characteristics	No. of Patients	Univariate Analysis		Multivariate Analysis	
		5-Year DFS Rate (%)	*p*-Value	HR (95% CI)	*p*-Value
Age			0.34		
<60 years	111 (30.9%)	54.7			
≥60 years	248 (69.1%)	51.1			
Sex			0.25		
Male	238 (66.3%)	49.2			
Female	121 (33.7%)	58.1			
Pathological T status			<0.001 *		
1 + 2	52 (44.4%)	74.7			
3 + 4	65 (55.6%)	34.5		2.08 (1.36–3.17)	0.001 *
Pathological N status			<0.001 *		
0	54 (46.2%)	71.0			
1 + 2 + 3	63 (53.8%)	35.3			
Pathological tumor stage			<0.001 *		
I + II	64 (54.7%)	70.9			
III	53 (45.3%)	25.9		1.62 (1.02–2.59)	0.043 *
Grade			0.049 *		
1 + 2	37 (31.6%)	58.5			
3	80 (68.4%)	48.2			
Total gastrectomy			0.014 *		
Yes	24 (20.5%)	37.6			
No	93 (79.5%)	54.3			
Perineural invasion			<0.001 *		
Yes	65 (55.6%)	32.0			
No	52 (44.4%)	71.1			
Lymphovascular invasion			<0.001 *		
Yes	72 (61.5%)	39.4			
No	45 (38.5%)	68.3			
Lymph node ratio			<0.001 *		
<25%	81 (69.2%)	64.1			
≥25%	36 (30.8%)	15.6		2.22 (1.49–3.32)	<0.001 *
AKR1B10			0.001 *		
Overexpression	117 (32.6%)	40.5		1.81 (1.32–2.47)	<0.001 *
Low expression	242 (67.4%)	57.8			

* Statistically significant.

**Table 4:** Univariate and multivariate analyses of overall survival (OS) in 359 gastric adenocarcinoma patients receiving gastrectomy.

Characteristics	No. of Patients	Univariate Analysis		Multivariate Analysis	
		5-Year OS Rate (%)	*p*-Value	HR (95% CI)	*p*-Value
Age			0.33		
<60 years	111 (30.9%)	60.7			
≥60 years	248 (69.1%)	56.1			
Sex			0.19		
Male	238 (66.3%)	55.6			
Female	121 (33.7%)	61.4			
Pathological T status			<0.001 *		
1 + 2	52 (44.4%)	79.4			
3 + 4	65 (55.6%)	40.2		1.97 (1.24–3.12)	0.004 *
Pathological N status			<0.001 *		
0	54 (46.2%)	76.5			
1 + 2 + 3	63 (53.8%)	40.3			
Pathological tumor stage			<0.001 *		
I + II	64 (54.7%)	77.9			
III	53 (45.3%)	28.2		1.83 (1.12–3.00)	0.017 *
Grade			0.08		
1 + 2	37 (31.6%)	63.4			
3	80 (68.4%)	53.8			
Total gastrectomy			0.006 *		
Yes	24 (20.5%)	41.9			
No	93 (79.5%)	59.7			
Perineural invasion			<0.001 *		
Yes	65 (55.6%)	35.5			
No	52 (44.4%)	77.5			
Lymphovascular invasion			<0.001 *		
Yes	72 (61.5%)	43.5			
No	45 (38.5%)	74.9			
Lymph node ratio			<0.001 *		
<25%	81 (69.2%)	70.0			
≥25%	36 (30.8%)	18.2		2.23 (1.48–3.35)	<0.001 *
AKR1B10			<0.001 *		
Overexpression	117 (32.6%)	44.7		1.93 (1.39–2.67)	<0.001 *
Low expression	242 (67.4%)	63.5			

* Statistically significant.

## Data Availability

The data presented in this study are available in this article.
